# Retraction: Application of biosensors in cancers, an overview

**DOI:** 10.3389/fbioe.2023.1343822

**Published:** 2023-12-05

**Authors:** Frontiers Editorial Office

“Following publication, concerns were raised regarding the contribution of the author of this article. Our investigation, conducted in accordance with Frontiers policies, confirmed a serious breach of our authorship policies and of publication ethics; the article is therefore retracted.

The retraction was approved by the Chief Editors of Bioengineering and Biotechnology and the Chief Executive Editor of Frontiers. The author does not agree to this retraction.”

